# Treatment patterns and steroid dose for adult minimal change disease relapses: A retrospective cohort study

**DOI:** 10.1371/journal.pone.0199228

**Published:** 2018-06-18

**Authors:** Takaya Ozeki, Masahiko Ando, Makoto Yamaguchi, Takayuki Katsuno, Sawako Kato, Yoshinari Yasuda, Naotake Tsuboi, Shoichi Maruyama

**Affiliations:** 1 Department of Nephrology, Nagoya University Graduate School of Medicine, Nagoya, Japan; 2 Center for Advanced Medicine and Clinical Research, Nagoya University Hospital, Nagoya, Japan; 3 Department of Nephrology, Yokkaichi Municipal Hospital, Yokkaichi, Japan; Kawasaki Ika Daigaku, JAPAN

## Abstract

**Background:**

In patients with adult minimal change disease (MCD), proteinuria relapse is a problem to solve. However, the optimal relapse treatment regimen remains unclear regarding steroid dose. We described the treatment pattern of adult MCD patients and evaluated the appropriate steroid dose for relapse treatment.

**Methods:**

This retrospective multicenter cohort study included 192 patients with adult biopsy-proven MCD from 14 hospitals in Japan. The prescription pattern of immunosuppressive drugs in relapse was reviewed. To assess the association between steroid dose used for relapse and subsequent outcomes, data of patients with tapered prednisolone (PSL) dosage to <10 mg/day before the first relapse in whom the dose was subsequently increased to ≥10 mg/day were extracted and assigned to the High-PSL or Low-PSL groups, based on the median dose of 20 mg/day. Multivariate Cox proportional hazard model and propensity score analysis with multiple imputations were conducted to compare their clinical course.

**Results:**

During a median observation period of 37.6 months, 186/192 (96.9%) patients achieved complete remission (CR) and 100 (52.1%) relapsed. The median urinary protein level at the first relapse was 3.12 g/gCr or g/day. The proportion of non-steroidal immunosuppressant use increased with relapses; cyclosporine was the most common. No significant differences were found in the second relapse, frequent relapses, or adverse events between High-PSL (n = 34) and Low-PSL (n = 36) groups. A multivariate Cox proportional hazard model revealed that the hazard ratios adjusted with propensity score for the second relapse were 0.94 (High-PSL vs. Low-PSL; 95% confidence interval, 0.42–2.10; P = 0.88) and 0.82 (PSL dose per 10 mg/day; 95% confidence interval, 0.58–1.16; P = 0.25).

**Conclusions:**

Among patients in CR with PSL dose <10 mg/day, higher steroid dose (PSL >20 mg/day) was not associated with favorable outcomes after the first relapse as compared to lower dose (10–20 mg/day).

## Introduction

Minimal change disease (MCD) is a major cause of idiopathic nephrotic syndrome, characterized by intense proteinuria leading to edema and intravascular volume depletion [1]. Although the incidence of MCD is dominant in children, MCD is also common in the adult population. Previous studies have reported that biopsy confirmed-MCD occurs in about 15–40% of adult patients with nephrotic syndrome [2–8]. According to several observational studies, over 90% of adult patients with MCD achieve complete remission (CR) by immunosuppressive treatment, but 40–70% of patients experience at least one relapse during their clinical course [9–17]. Although relapses are considered a serious problem in the clinical course of MCD, there are currently no clear recommendations for treatment of relapse in the guidelines and the practice pattern of MCD relapse treatment has not been described. Furthermore, corticosteroids are key drugs for adult patients with MCD, but optimal steroid dose for relapse is still unclear. There are differences between Kidney Disease Improving Global Outcomes (KDIGO) guidelines and Japanese guidelines [18,19] in the recommendations for treatment of MCD relapse. KDIGO guidelines suggest the same steroid dose for relapse as the initial dose (0.8–1.0 mg/kg/day); conversely, Japanese guidelines are conflicting and indicate the same dose as the initial treatment or a reduced dose of 20–30 mg/day for relapse. Recently, an observational study in pediatric patients reported that low dose and short-term steroid administration in the treatment for relapse of steroid sensitive nephrotic syndrome improved patients’ quality of life during their clinical course without changing the relapse rate [20].

The aim of this study was to describe the treatment pattern for relapse of adult MCD and to identify the ideal steroid dose for treatment of relapsed adult MCD.

## Materials and methods

This study was a retrospective, multi-center cohort study comprising 14 nephrology centers, including Nagoya University Hospital and its affiliated hospitals (Chubu Rosai Hospital, Toyohashi Municipal Hospital, Japanese Red Cross Nagoya Daiichi Hospital, Anjo Kosei Hospital, Ogaki Municipal Hospital, Kasugai Municipal Hospital, Ichinomiya Municipal Hospital, Handa City Hospital, Nagoya Kyoritsu Hospital, Tosei General Hospital, Konan Kosei Hospital, Yokkaichi Municipal Hospital, and Gifu Prefectural Tajimi Hospital).

Patients aged over 20 years old with a first-time episode of nephrotic syndrome, and diagnosed with biopsy-proven MCD were included in the study. The enrollment period was from January 2005 to December 2013 and the follow-up period was from January 2005 to December 2014. The exclusion criteria were as follows: (i) missing medical records, (ii) concomitant kidney diseases, (iii) secondary MCD [21], (iv) concomitant malignancies (under treatment), (v) other disorders which required immunosuppressive therapies, and (vi) spontaneous remission or no immunosuppressive therapies.

### Data collection

The clinical information was collected retrospectively from medical records.

#### Baseline characteristics

The time of immunosuppressive treatment initiation was used as baseline. Baseline characteristics included age, sex, body mass index, systolic/diastolic blood pressure, and prescription of antihypertensive drugs, including angiotensin-converting enzyme inhibitors, angiotensin II receptor blockers, calcium channel blockers, and β-blockers. Baseline laboratory data included serum total protein, albumin, total cholesterol, IgG, C3, and C4 levels. Serum creatinine (Scr) concentration was also obtained, and glomerular filtration rate (GFR; estimated by using the equation for Japanese: eGFR [mL/min/1.73 m^2^] = 194 × Scr^-1.094^ × Age^-0.287^ × 0.739 [if female])[22]. Urinary occult blood level, and 24-hour urinary protein excretion (g/day) or urinary protein-creatinine ratio (g/gCr) were also collected.

#### Initial treatment and induction of remission

Data regarding initial treatment and induction of remission were collected as follows: initial prednisolone (PSL) dose, methylprednisolone (mPSL) pulse, combined other non-steroidal immunosuppressive agents (ISA), 25% human albumin administration, temporary hemodialysis, low density lipoprotein apheresis. Moreover, achievement of the first remission and time to first remission were also collected.

#### Incidence of relapse and treatment for relapse

The following data regarding relapses of proteinuria were collected: incidence of relapses, total number of relapses, duration from remission to relapse (relapse-free days), and the details of treatment at each relapse. Clinical and laboratory findings at the first relapse, such as serum albumin, creatinine, total cholesterol, and the urinary protein-creatinine ratio, were also collected.

#### Complications of MCD and adverse events from immunosuppressive treatment

Complications or adverse events in clinical course included death, infection requiring admission, malignancy, thrombosis, cardiovascular disease requiring admission, cerebrovascular disease requiring admission, de novo diabetes mellitus, femoral head osteonecrosis, peptic ulcer disease, 30% and 50% decline of eGFR, and initiation of maintenance hemodialysis. In order to estimate the basal value of Scr before the onset of MCD, the basis of eGFR decline during the entire observation period was obtained from the minimal value at the initiation of immunosuppressive treatment as the baseline and after 1, 2, 6, and 12 months from the baseline [23].

### Definitions

CR was defined as the reduction of proteinuria to <0.3 g/day, <0.3 g/gCr, and/or a negative result for urinary protein on dipstick test. Relapse was defined as increasing urinary protein to ≥0.3 g/day, ≥0.3 g/gCr, and/or ≥1+ on dipstick test with strengthening of immunosuppressive treatment. Because of the retrospective study design, the timing of treatment strengthening depended on the decision of attending nephrologists.

### Outcome measurement

#### Analysis 1: Incidence and treatment pattern of MCD relapses

To describe the practice pattern for management of relapses in adult patients with MCD, the following details of immunosuppressive treatment were evaluated: the dose of steroid and combined non-steroidal ISA used at every relapse.

#### Analysis 2: Optimal steroid dose for relapse

In addition, we evaluated the optimal steroid dose for relapse. The outcomes of interest were the time of the second relapse from the first relapse and the incidence of frequent relapses and adverse events after the first relapse.

### Ethics

All patients provided written informed consent. This study was approved by the respective ethics committees of all 14 centers listed above in agreement with the Declaration of Helsinki (approval number: 2010-1135-4).

### Statistics

#### Analysis 1: Incidence and treatment pattern of MCD relapses

Clinical characteristics of included patients were expressed in medians/interquartile ranges (IQR) and frequency number/percentages.

#### Analysis 2: Optimal steroid dose for relapse

To evaluate the optimal steroid dose for relapse, we extracted data from the cohort cases fulfilling the following conditions: (i) the first relapse occurred with a PSL dose <10 mg/day and (ii) immunosuppressive therapy strengthened by increasing PSL dose to ≥10 mg/day after the first relapse. Patients with PSL <10 mg/day or with strengthened non-steroidal immunosuppressants treatment without increasing PSL dose for the first relapse were excluded from this analysis because the evaluation of the impact of steroid dose increase was considered difficult in them. Eligible cases were divided into two groups based on the median value of PSL dose for the first relapse (High-PSL and Low-PSL groups). We used the Student’s t-test or Mann-Whitney’s U test to compare the continuous variables and Chi-squared test or Fischer’s exact test to compare the proportions of categorical variables between the groups. To compare the effect of the difference in PSL dose in relapse treatment, we used a Cox regression model. Baseline variables with P <0.15 in univariate analysis were included in the multivariable models. Proportional hazards assumption for covariates was tested using scaled Schoenfeld residuals.

To adjust for imbalances in patient characteristics at the first relapse between the groups, we calculated a propensity score (PS) to estimate the probability of receiving a higher PSL dose at first relapse. The PS was obtained using a multivariate logistic regression model including baseline age (10 year-intervals), sex, serum albumin and creatinine concentrations at the first relapse, urinary protein level at the first relapse with logarithmic transformation, relapse-free days (10-day intervals), days to achieve CR in initial treatment, PSL dose before the first relapse, and non-steroidal ISA use before the first relapse. Before PS calculation, multiple imputation (MI) analysis was performed to adjust for the missing data and 20 multiply imputed datasets were created using the Markov chain Monte Carlo method. After PS calculation, PS were inserted into the Cox proportional hazard model as a variable [24] with steroid dose at the first relapse in each imputed dataset and hazard ratios were merged by Rubin’s method. To confirm the robustness of the result, we repeated same analysis after trimming the units with PS outside the range of [0.1, 0.9] [25].

All statistical analyses were conducted using STATA version 14.0 (StataCorp LLC, College Station, TX, USA) and SAS version 9.4 (SAS Institute, Cary, NC, USA). The statistical significance level was set at P <0.05.

## Results

### Analysis 1: Incidence and treatment pattern of MCD relapses

**Fig 1:**
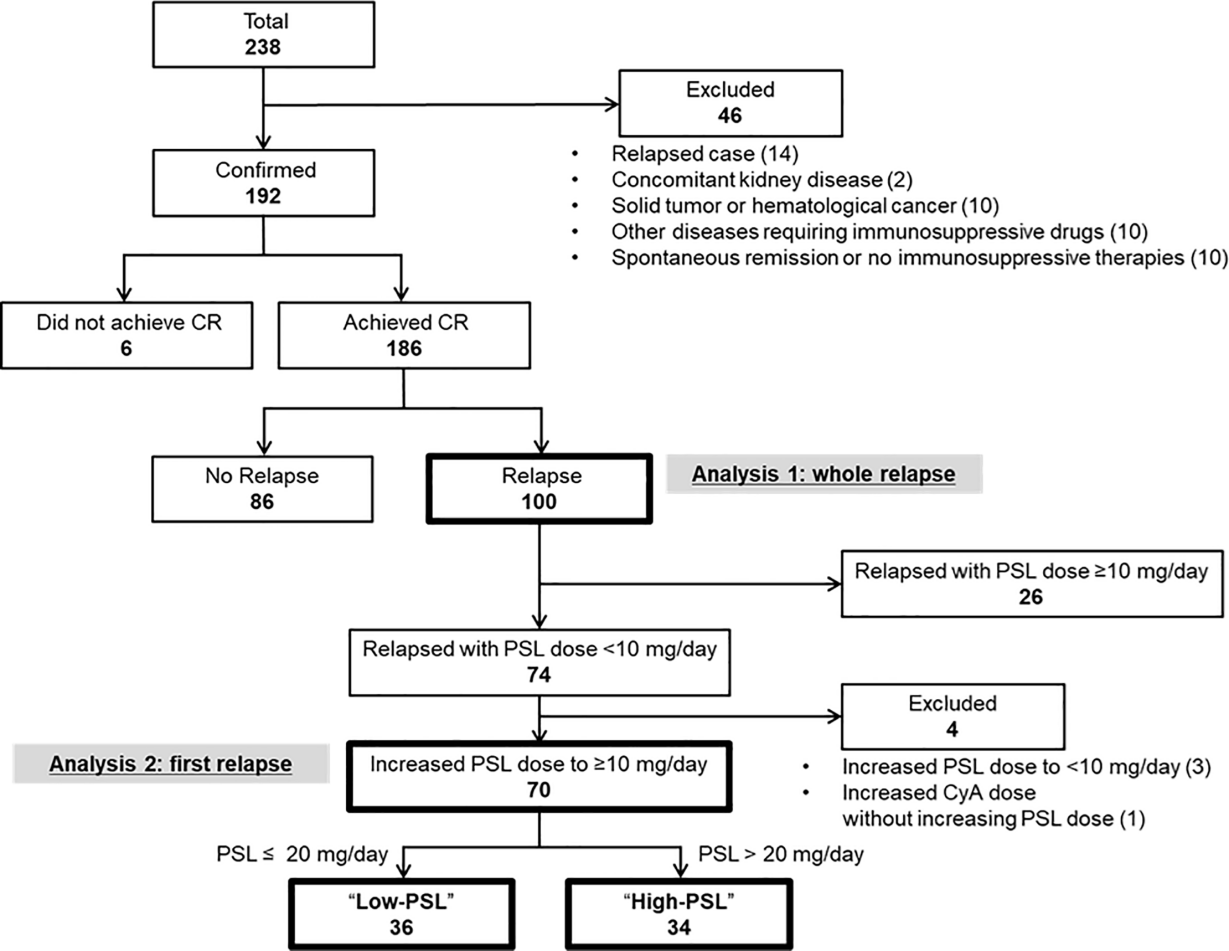
Overview of this study. Of the 238 adult patients with minimal change disease in the cohort, 192 were eligible for this study. Analysis 1: For the 100 identified patients with relapse, treatment regimens at every relapse were reviewed. Analysis 2: To evaluate the association between steroid dose and subsequent outcomes, patients who fulfilled the criteria as indicated in the flow chart were divided into two groups: “High-PSL” and “Low-PSL.” Abbreviations: CR; complete remission, PSL; prednisolone; CyA, cyclosporine A.

**Fig 2:**
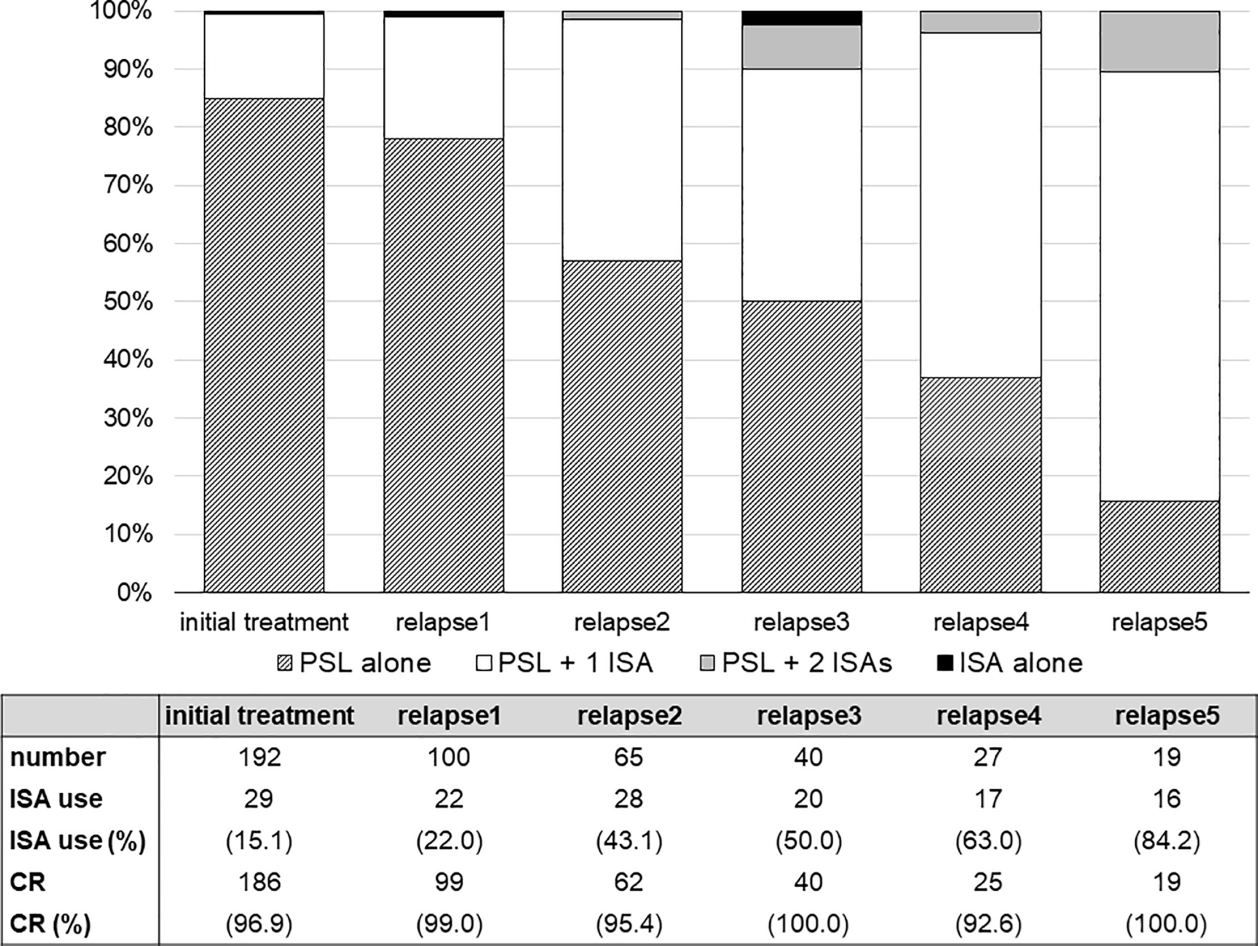
Demographic pattern of immunosuppressive treatment for adult minimal change disease relapses. Proportion of steroid and other immunosuppressive drugs use in treatment of adult MCD from initial treatment to fifth relapse are shown in bar-graphs. The details about types of used non-steroidal immunosuppressants are summarized in S4 Table. Abbreviations: MCD, minimal change disease; PSL, prednisolone; ISA, non-steroidal immunosuppressive agents; CR, complete remission.

**Fig 3:**
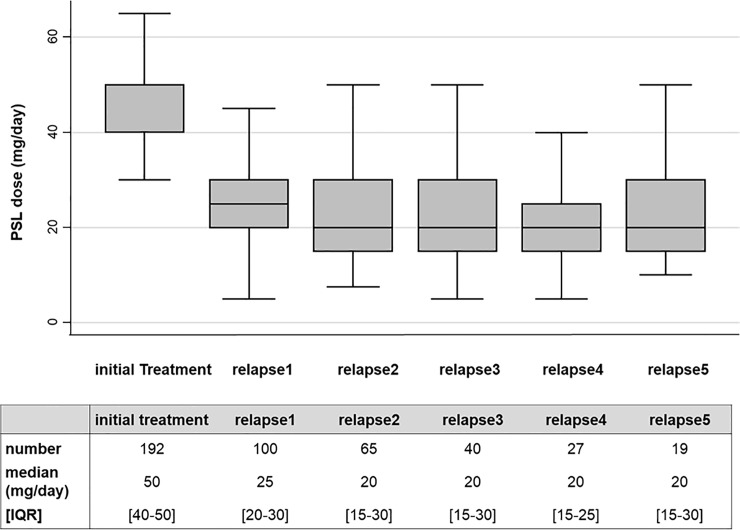
Prednisolone dose for initial treatment and relapses. Steroid dose in treatment of adult MCD from initial treatment to fifth relapse is shown in box-plots. The dosage at the start of each treatment was selected and the dosage of additional increasing until complete remission was excluded. Abbreviations: PSL, prednisolone; IQR, interquartile range.

Patients’ selection and clinical course of present study are summarized in Fig 1. Among 238 adult patients with MCD, 192 patients were eligible for this study. Median duration of follow-up was 37.6 months. The baseline clinical and laboratory data of patients and the details of initial treatment, profiles of relapse and adverse events are summarized in S1–S5 Tables. One hundred eighty-six patients (96.9%) achieved CR within a median of 14 days. The clinical and laboratory data at the first relapse of relapsed cases are shown in S3 Table. One hundred (52.1%) patients experienced at least one relapse (up to a maximum of 15 relapses). Among the relapsed cases, 71/100 were infrequent relapses and 29/100 were frequent relapses. Median steroid dose before the first relapse was 5.0 mg/day. At the first relapse, the median value of proteinuria was 3.12 g/gCr or g/day, and 45.0% of patients with relapse showed minor relapse with sub-nephrotic proteinuria (<3.5 g/day). The details of the treatment are also shown in Figs 2 and 3. As initial therapy, all patients were treated with corticosteroid and the percentages of other non-steroidal ISA use increased as the number of relapses increased. The details of the second- or third-line ISA used are summarized in S4 Table. Cyclosporine was the most common drug that was combined with steroids in our cohort. Cyclophosphamide was administered in two patients, and no patient received rituximab therapy. For initial therapy, the median dose of PSL was 50 mg/day. The median dose of PSL on the first, second, third, fourth, fifth relapse treatment was 25, 20, 20, 20, and 20 mg/day, respectively.

### Analysis 2: Optimal steroid dose on relapse

**Fig 4:**
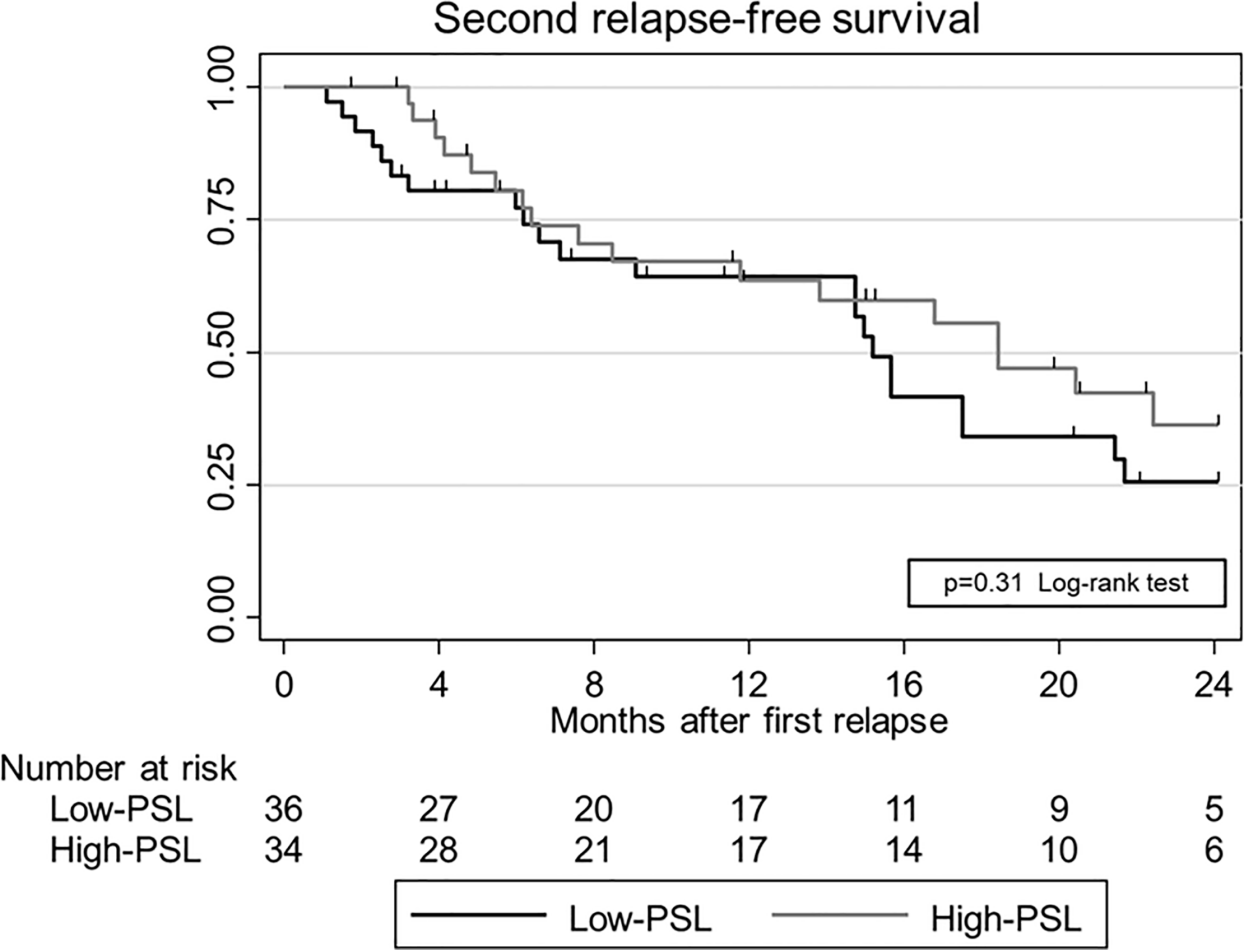
Second relapse-free survival of minimal change disease after first relapse. Second relapse-free survival after first relapse in the High-prednisolone (PSL) group (n = 34) and Low-PSL group (n = 36) were calculated using the Kaplan–Meier method and compared by log-rank test. Abbreviations: MCD, minimal change disease; PSL, prednisolone.

Of the 100 patients with first relapse, 74 relapsed on PSL dose <10 mg/day. Subsequently, 70 patients were treated by PSL with ≥10 mg/day and were included in this analysis: 36 patients in the Low-PSL group and 34 patients in the High-PSL group (Fig 1). Clinical and laboratory characteristics of each group are shown in Table 1. At the time of first relapse, these two groups differed significantly in their serum albumin and urinary protein levels. In contrast, immunosuppressive treatment for relapse, except for steroid dose for mPSL pulse and/or combination of other non-steroidal ISA, was not significantly different between the two groups. All patients, except one from Analysis 2, achieved CR again after the first relapse (Table 2). During the clinical course after the first relapse, no significant differences were observed in incidence of the second relapse, frequent relapse, or adverse events (Table 2, Fig 4). Kaplan-Meier curves estimating the relapse-free survival until second relapse are shown in Fig 4. The log-rank test showed no significant difference between the two groups (P = 0.31). Univariate and multivariate analysis in a Cox proportional hazard model for second relapse are summarized in Tables 3 and 4. Univariate analysis revealed that only relapse-free time before the first relapse was significantly associated with a second relapse. In multivariate analysis, the adjusted hazard ratio (HR) for second relapse was not significantly different in the High-PSL versus Low-PSL group (HR = 0.72; 95% CI = 0.21–2.48; P = 0.61) and in increasing PSL dose per 10 mg/day (HR = 0.81; 95% CI = 0.53–1.24; P = 0.33).

**Table 1:** Clinical characteristics of the patients at first relapse.

**Patient Characteristics at first relapse**	**Low-PSL** **(n = 36)**		**High-PSL** **(n = 34)**		**P-value**
Age	56	[40–66]	47	[36–59]	0.089
Sex, male	25	(69.4)	21	(61.8)	0.50
Relapse free days, days	374	[222–581]	498	[327–737]	0.084
PSL dose before first relapse, mg/day	5	[1.1–5.5]	0	[0–2.5]	0.003
ISA usage before first relapse	4	(11.1)	0	0.0	0.115
	CyA (4)		-		
Albumin, g/dL	3.9	[3.5–4.3]	3.1	[2.6–3.6]	< 0.001
Creatinine, mg/dL	0.78	[0.68–1.07]	0.80	[0.69–0.94]	0.84
Total Cholesterol, mg/dL	209	[186–245]	293	[231–327]	< 0.001
Urinary protein, g/24h, g/gCr	1.27	[0.79–3.14]	4.90	[2.64–8.93]	< 0.001
**Treatment for first relapse**	**Low-PSL**		**High-PSL**		**P-value**
PSL dose after first relapse, mg	20	[15–20]	30	[30–40]	< 0.001
mPSL pulse	1	(2.8)	1	(2.9)	0.97
Combined ISA administration	5	(13.9)	3	(8.8)	0.71
Increased ISA dose	3	(8.3)	0	(0.0)	0.085
	CyA (3)		-		
Added on new ISA	2	(5.6)	3	(8.8)	0.60
	CyA (2)		CyA (3)		

Values are given as median [interquartile range] or number (percent).

Abbreviations: PSL, prednisolone; mPSL, methylprednisolone; ISA, non-steroidal immunosuppressive agents; CyA, cyclosporine

**Table 2:** Re-induction of complete remission, subsequent relapses and adverse events in relapsed cases.

Outcome		Low-PSL (n = 36)		High-PSL (n = 34)		P-value
**Remission**	CR of first relapse	35	(97.2)	34	(100.0)	1.00
	Days to achieve CR of first relapse*	21	[13–43]	14	[11–28]	0.172
**Subsequent** **Relapse**	Second relapse*	22	(62.9)	21	(61.8)	1.00
	Days to second relapse from baseline*, days	826.5	[433–949]	932.5	[535–1308]	0.32
	Days to second relapse from first relapse*, days	314	[122–575]	389	[147–682]	0.37
	Frequent relapse*	10	(28.6)	9	(26.5)	0.85
**Adverse events after** **first relapse**	Death	0	(0.0)	1	(2.9)	0.49
	Infection**	2	(5.6)	1	(2.9)	1.00
	Malignancy	2	(5.6)	0	(0.0)	0.49
	Thrombosis	0	(0.0)	1	(2.9)	0.49
	Cardiovascular disease**	2	(5.6)	0	(0.0)	0.49
	Cerebrovascular disease**	0	(0.0)	0	(0.0)	-
	de novo DM	1	(2.8)	2	(5.7)	0.61
	Femoral head osteonecrosis	1	(2.8)	1	(5.7)	1.00
	Peptic ulcer disease	2	(5.6)	1	(2.9)	1.00
	eGFR 30% decline	3	(8.3)	1	(2.9)	0.61
	eGFR 50% decline	0	(0.0)	0	(0.0)	-
	Maintenance HD	0	(0.0)	0	(0.0)	-

Values are given as median [interquartile range] or number (percent).

*Excluding one patient in Low-PSL group who was censored without remission after strengthened immunosuppressive therapy for first relapse.

**required hospitalization

Abbreviations: PSL, prednisolone; CR, complete remission; DM, diabetes mellitus; eGFR, estimated glomerular filtration rate; HD, hemodialysis

**Table 3:** Predictors of second relapse.

Predictors	Univariate model		
	HR	[95%CI]	P-value
Age at first relapse (every 10 years)	0.87	[0.71–1.06]	0.178
Sex (Male)	1.16	[0.60–2.27]	0.66
Relapse-free before first relapse (every 10 days)	0.98	[0.97–0.99]	0.003
PSL dose before relapse	1.07	[0.97–1.18]	0.161
Combined ISA usage before first relapse	0.52	[0.12–2.17]	0.37
Log_UTP at first relapse	0.95	[0.67–1.35]	0.78
Serum albumin at first relapse	1.41	[0.92–2.16]	0.113
Serum creatinine at first relapse	0.32	[0.77–1.30]	0.111
PSL after first relapse (High-PSL)	0.72	[0.38–1.36]	0.31
PSL dose after first relapse (every 10 mg/day)	0.80	[0.59–1.08]	0.139

Abbreviations: PSL, prednisolone; ISA, non-steroidal immunosuppressive agents; UTP, urinary protein level

**Table 4:** PSL dose for the treatment of first relapse as a predictor of second relapse.

**PSL dose as a categorical variable** **(High-PSL versus Low-PSL)**	**HR**	**[95% CI]**	**P-value**
Univariate	0.80	[0.44–1.47]	0.48
Multivariate ^a)^	0.72	[0.32–1.65]	0.44
Multivariate with PS ^b)^ after MI ^c)^	0.94	[0.42–2.10]	0.88
Multivariate with trimmed PS after MI	0.87	[0.39–1.94]	0.73
**PSL dose as a continuous variable** **(every 10mg/day)**	**HR**	**[95%CI]**	**P-value**
Univariate	0.81	[0.62–1.07]	0.136
Multivariate ^a)^	0.85	[0.57–1.25]	0.40
Multivariate with PS ^b)^ after MI ^c)^	0.82	[0.58–1.16]	0.25
Multivariate with trimmed PS after MI	0.83	[0.55–1.22]	0.34

Abbreviations: HR, hazard ratio; CI, confidence interval; PSL, prednisolone; PS, propensity score; MI, multiple imputation

a) adjusted for relapse-free days (every 10 days), serum albumin at first relapse, serum creatinine at first relapse, and urinary protein level at first relapse

b) Propensity score was obtained from the covariates; age (every 10 years), sex (male), relapse-free days (every 10 days), PSL dose before relapse, urinary protein level at first relapse (log transformation) with MI, serum albumin at first relapse with MI, serum creatinine at first relapse with MI, and enhance non-steroid immunosuppressive treatment at the timing of first relapse

c) Multiple imputation was performed for serum albumin, creatinine, and urinary protein (log transformation) using regression model from age (every 10 years), sex (male), relapse-free days (every 10 days), PSL dose before relapse, and non-steroid immunosuppressive agent usage before relapse

We also performed multivariate analysis combined with PS and MI analysis. In obtaining PS, 15 of 70 eligible patients (20%) had missing values among the variables used for PS calculation. After imputed missing values by MI, PS was obtained and then the scores were added to the previous multivariate model as a covariate. The merged HR in the PS-based model revealed that the steroid dose after the first relapse did not significantly affect the occurrence of a second relapse (High-PSL vs. Low-PSL: HR = 0.94; 95% CI = 0.42–2.10; P = 0.88) (PSL dose per 10 mg/day: HR = 0.82; 95% CI = 0.58–1.16; P = 0.25). Moreover, the same analysis with a trimmed PS also revealed that the steroid dose at the first relapse was not significantly associated with a second relapse (High-PSL vs. Low-PSL: HR = 0.87; 95% CI = 0.39–1.94; P = 0.73) (PSL dose per 10 mg/day: HR = 0.83; 95% CI = 0.55–1.22; P = 0.34).

## Discussion

In this study, we investigated the clinical course of adult MCD, especially from the viewpoint of relapse of proteinuria. This study has several strong points. The number of patients included in our cohort was 192, which is one of the largest cohorts of adult patients with MCD reported to date. In addition, this is the first study that describes the treatment pattern, including the tendency and details of non-steroidal immunosuppressive drugs, and optimal steroid dose used in patients with MCD relapse.

As shown in Analysis 1, the overall relapse rate was 52.1% (100/192) in our cohort. And 45.0% (45/100) cases showed relapse with sub-nephrotic proteinuria (<3.5 g/gCr). In Japanese guidelines, relapse is defined as the condition that urine protein ≥1 g/gCr runs or ≥(2+) continues 2–3 times in a row [19]. Cyclosporine was the most common non-steroidal ISA used in patients with relapse, but cyclophosphamide, recommended by the KGIDO guidelines, was prescribed for only two patients in our cohort. Some patients with multiple relapses were treated with steroid plus two different non-steroidal ISAs in combination, but no clinical trials to support this practice are currently available. Recently, an increasing number of reports on the effectiveness of the anti-CD20 monoclonal antibody rituximab for refractory cases of adult MCD have been reported [26–29]. No patient received rituximab therapy because it had not been approved in Japan in this study period.

Subsequently, the optimal steroid dose for relapse was analyzed (Analysis 2). On univariate analysis, the steroid dose increased at the first relapse was not associated with a second relapse. While patient age [15] or kidney function [30] at initial treatment were previously reported as associated factors of the first relapse, we did not find any association between patient age or kidney function at the first relapse and second relapse in this study. Multivariate analysis revealed that increased steroid dose at the first relapse was not significantly associated with second relapse. These results suggested that relapses in adult MCD might be successfully controlled by a low-dose PSL of approximately 20 mg/day even taking into account the amount of proteinuria or prior combination of immunosuppressants. Moreover, a high-dose PSL as recommended by the KDIGO guidelines may not be necessary, if the relapse occurred on a PSL dose of less than 10 mg/day. Although the patients who were included in Analysis 2 represented only 36.5% of the total, the observed number of second relapses in this study yielded a statistical power of more than 75% for detecting HR of 0.5 at the 5% significance level in both High-PSL and Low-PSL patients.

Our data contained the patients who treated as a relapse with very-mild proteinuria. For eliminating the effect of such patients, we conducted same analysis after excluding the patients whose urinary protein level was <1.0 g/gCr at first relapse (n = 11; 10 in the Low-PSL group, one in the High-PSL group). As shown in S6 Table, we obtained similar results from the original data.

Does low-dose steroid treatment increase the requirement of additional treatment for adult patients with MCD relapse? In Analysis 2, seven patients (20.5%) from High-PSL group and eight patients (22.2%) from Low-PSL group required additional strengthening of their treatment (i.e., further increase of the PSL dose, addition of mPSL pulse, and/or new ISAs) after the initial setting of the PSL dose at the initiation of treatment for the first relapse. Defining such conditions as “additional treatment,” the rates were not significantly different between the groups, (P = 0.87). This result suggested that low-dose steroid could be applied to all MCD relapse cases. However, it was difficult to predict the cases that would require additional treatment based on clinical information at the first relapse (S7 Table). This unclear result might derive from the fact that the pathophysiology of MCD and the pharmacological mechanism of steroids in MCD treatment have not been sufficiently elucidated to date [1].

MCD has been considered to develop from a series of podocyte injuries [31,32] and humoral factors (e.g., CD80 [33], hemopexin [34] and others) and may be associated with the onset of proteinuria. However, responsible biomarkers predictive of MCD have not been identified [35]. In the future, if new biomarkers predicting disease activity or therapeutic reactivity of MCD are identified, it may help to optimize the steroid dose in relapse treatment for each patient.

Several limitations of this study should be acknowledged. First, the definition of relapse differed from that of other studies, and patients with sub-nephrotic proteinuria were included as relapse cases in our study, but we consider that this study showed the real-world evidence. Second, in Analysis 2, we examined only those patients who were treated with PSL of <10 mg/day at the first relapse. Third, because of retrospective design, we did not have any regular protocols of steroid administration for the relapsed patients. It is difficult to evaluate the impact of the difference of steroid dose for relapse treatment including tapering rate or cumulative dosage of PSL in this study. Although we used statistical techniques, such as multivariate analysis and PS to adjust for confounders and imbalances between the two groups, any potential unmeasured confounding factors may not have been considered in the analysis. A randomized-controlled trial is required to confirm our results.

In conclusion, discrepancies still exist between recommendations in the KDIGO guidelines and current practice in the treatment of adult MCD relapse in Japan. Clinical outcomes of patients with MCD who were treated with lower dose (10–20 mg/day) steroid therapy were not different from those of patients treated with higher steroid dose (>20 mg/day). The present study suggests that higher steroid dose may not be necessary for the treatment of adult MCD relapse.

## Supporting information

S1 Table Clinical characteristics of all cases in this study at initiation of immunosuppressive treatment. (DOCX)

S2 Table Initial treatment and induction of complete remission in all cases. (DOCX)

S3 Table Incidence of relapse in all cases and treatment profiles for first relapse. (DOCX)

S4 Table Type of non-steroidal immunosuppressive agents in all cases. (DOCX)

S5 Table Complications and adverse events in all cases. (DOCX)

S6 Table PSL dose for the relapse treatment of first relapse as a predictor of second relapse: additional analysis after excluding the patients with very-mild proteinuria. (DOCX)

S7 Table Patient characteristics with or without additional treatment at first relapse. (DOCX)
