# Supplementary material for: Treatment patterns and steroid dose for adult minimal change disease relapses: A retrospective cohort study
Source: PLoS One. 2018 Jun 18;13(6):e0199228. doi: 10.1371/journal.pone.0199228 (PMC6005527; doi:10.1371/journal.pone.0199228)
Supplement: S1 Table — (DOCX) [file pone.0199228.s001.docx]

**S1 Table. Clinical characteristics of all cases in this study at initiation of immunosuppressive treatment.**

| **All cases (n=192)** | | **Median, *n*** | **[IQR], (%)** | **Min, Max** |
| --- | --- | --- | --- | --- |
| **Patient**  **characteristics** | Age | 50.0 | [ 35.5-65] | 20, 83 |
|  | Sex, male | 113 | (58.9) |  |
|  | Height, cm | 162 | [156-168] | 142, 180 |
|  | Body weight, kg | 63.2 | [56.0-71.3] | 37.8, 126.2 |
|  | Body Mass Index | 24.3 | [22.0-27.1] | 15.1, 42.7 |
|  | Systolic blood pressure, mmHg | 120 | [107-133] | 86, 198 |
|  | Diastolic blood pressure, mmHg | 73 | [66-82] | 38, 111 |
|  | Diabetes mellitus | 12 | (6.3) |  |
|  | Hypertension | 63 | (32.8) |  |
|  | Anti-hypertensive drugs | 51 | (26.8) |  |
|  | ACE-I/ARB | 36/51 | (70.6) |  |
|  | Diuretics | 121 | (63.7) |  |
|  | Statins | 56 | (29.5) |  |
|  | Smoking status |  |  |  |
|  | Never | 72 | (37.5) |  |
|  | Ex-smoker | 42 | (21.9) |  |
|  | Current smoker | 52 | (27.1) |  |
|  | No data | 26 | (13.5) |  |
| **Laboratory data** | Total protein, g/dL | 4.1 | [3.7-4.7] | 2.7, 6.6 |
|  | Albumin, g/dL | 1.5 | [1.2-1.9] | 0.5, 2.9 |
|  | Creatinine, mg/dL | 0.95 | [0.71-1.24] | 0.48, 7.40 |
|  | eGFR, mL/min/1.73m2 | 63 | [46-81] | 6, 126 |
|  | Total cholesterol, mg/dL | 410 | [315-496] | 205, 736 |
|  | IgG, mg/dL | 554 | [394-735] | 115, 1851 |
|  | C3, mg/dL | 134 | [120.5-158.5] | 66, 329 |
|  | C4, mg/dL | 39 | [32-45] | 7, 209 |
|  | Urinary protein level, g/24h, g/gCr) | 7.15 | [4.87-10.36] | 0.95, 31.88 |
|  | Urinary occult blood |  |  |  |
|  | (-) | 18 | (9.4) |  |
|  | (+/-) | 31 | (16.1) |  |
|  | (1+) | 44 | (23.0) |  |
|  | (2+) | 54 | (28.1) |  |
|  | (3+) | 41 | (21.3) |  |
|  | No data | 4 | (2.1) |  |

Abbreviations: ACE-I, angiotensin-converting enzyme inhibitors; ARB, angiotensin II receptor blockers; eGFR, estimated glomerular filtration rate; IQR, interquartile range
