# Supplementary material for: Treatment patterns and steroid dose for adult minimal change disease relapses: A retrospective cohort study
Source: PLoS One. 2018 Jun 18;13(6):e0199228. doi: 10.1371/journal.pone.0199228 (PMC6005527; doi:10.1371/journal.pone.0199228)
Supplement: S2 Table — (DOCX) [file pone.0199228.s002.docx]

**S2 Table. Initial treatment and induction of complete remission in all cases.**

| **All cases (*n*=192)** | | **Median, *n*** | **[IQR], (%)** | **Min, Max** |
| --- | --- | --- | --- | --- |
| **Initial treatment** | mPSL pulse | 35 | (18.2) |  |
|  | PSL route, oral | 182 | (94.8) |  |
|  | Initial PSL dose, mg/day | 50 | [40-50] | 10, 80 |
|  | Initial PSL dose, mg/kg/day | 0.75 | [0.65-0.84] | 0.37, 1.17 |
|  | Combination of ISAs | 30 | (15.6) |  |
|  |  | CyA (28), MZR (1), CYC (1) | | |
|  | 25% albumin administration | 43 | (22.4) |  |
|  | Temporary HD | 17 | (8.9) |  |
|  | LDL-A | 6 | (3.1) |  |
| **First remission** | Complete remission | 186 | (96.9) |  |
|  | Time to complete remission, days | 14 | [10-25] | 4, 538 |
|  | Add new ISAs after remission | 4 | (2.1) |  |
|  |  | CyA (3), AZA (1) | | |

Abbreviations: mPSL, methylprednisolone; PSL, prednisolone; ISA, non-steroidal immunosuppressive agents; HD, hemodialysis; LDL-A, low density lipoprotein apheresis; CyA, cyclosporine; MZR, mizoribine; CYC, cyclophosphamide; AZA, azathioprine
