# Supplementary material for: Treatment patterns and steroid dose for adult minimal change disease relapses: A retrospective cohort study
Source: PLoS One. 2018 Jun 18;13(6):e0199228. doi: 10.1371/journal.pone.0199228 (PMC6005527; doi:10.1371/journal.pone.0199228)
Supplement: S3 Table — (DOCX) [file pone.0199228.s003.docx]

**S3 Table. Incidence of relapse in all cases and treatment profiles for first relapse.**

| **All cases (*n*=192)** | | **Median, *n*** | **[IQR], (%)** | **Min, Max** |
| --- | --- | --- | --- | --- |
| **Relapse** | Relapse | 100 | (52.1) |  |
|  | Number of whole relapse | 1 | [0-2] | 0, 15 |
|  | Days to first relapse from baseline, days | 362 | [197-586] | 27, 1765 |
| **Relapsed cases (n=101)** | | **Median, n** | **[IQR], (%)** | **Max, Min** |
| **Treatment before**  **first relapse** | Treatment before first relapse |  |  |  |
|  | PSL alone | 65 | (64.4) |  |
|  | PSL + ISA | 8 | (7.9) |  |
|  |  | CyA (8) | |  |
|  | ISA alone | 1 | (1.0) |  |
|  |  | CyA (1) | |  |
|  | none | 27 | (26.7) |  |
|  | PSL dose before first relapse, mg/day | 5 | [0–10] | 0, 40 |
| **Laboratory data**  **at first relapse** | Albumin, g/dL | 3.6 | [3.0-3.9] | 1.0, 5.0 |
|  | Creatinine, mg/dL | 0.77 | [0.64-0.95] | 0.44, 1.43 |
|  | Urinary protein level, g/24h, g/gCr | 3.12 | [1.34-6.40] | 0.30, 20.80 |
| **Treatment for**  **first relapse** | Increase PSL | 99 | (99.0) |  |
|  | PSL dose after first relapse, mg/day | 25 | [20-30] | 5, 60 |
|  | mPSL pulse | 8 | (8.0) |  |
|  | ISA |  |  |  |
|  | Increased dose | 5 | (5.0) |  |
|  |  | CyA(5) | |  |
|  | New drugs added | 15 | (15.0) |  |
|  |  | CyA(13), MZR(2) | |  |
|  | CR of first relapse | 98 | (98.0) |  |
|  | Time to re-induction of CR, days | 21 | [13-36] | 4, 496 |
| **Subsequent**  **relapse** | Second relapse | 65 | (65.0) |  |
|  | Frequent relapse | 29 | (29.0) |  |

Abbreviations: PSL, prednisolone; ISA, non-steroidal immunosuppressive agents; mPSL, methylprednisolone; CyA, cyclosporine, MZR, mizoribine; CR, complete remission
