# Supplementary material for: Treatment patterns and steroid dose for adult minimal change disease relapses: A retrospective cohort study
Source: PLoS One. 2018 Jun 18;13(6):e0199228. doi: 10.1371/journal.pone.0199228 (PMC6005527; doi:10.1371/journal.pone.0199228)
Supplement: S4 Table — (DOCX) [file pone.0199228.s004.docx]

**S4 Table. Type of non-steroidal immunosuppressive agents in all cases.**

| **Number of relapse** | **n at risk** | **ISA** | **(%)** | **category** | **n** | **Type (*n*)** |
| --- | --- | --- | --- | --- | --- | --- |
| Initial treatment | 192 | 29 | (15.1) |  |  | CyA (27), MZR (1), CYC (1) |
|  |  |  |  | PSL + 1 ISA | 28 |  |
|  |  |  |  |  |  |  |
|  |  |  |  | ISA alone | 1 | CyA (1) |
| Relapse 1 | 100 | 22 | (22.0) |  |  |  |
|  |  |  |  | PSL + 1 ISA | 21 | CyA (20), MZR (1) |
|  |  |  |  |  |  |  |
|  |  |  |  | ISA alone | 1 | CyA (1) |
| Relapse 2 | 65 | 28 | (43.1) |  |  | CyA (24), MZR (2),  TAC (1) |
|  |  |  |  | PSL + 1 ISA | 27 |  |
|  |  |  |  |  |  |  |
|  |  |  |  | PSL + 2ISAs | 1 | CyA + MZR (1) |
| Relapse 3 | 40 | 20 | (50.0) | PSL + 1 ISA | 17 | CyA (17) |
|  |  |  |  | PSL + 2 ISAs | 3 | CyA + MZR (2) |
|  |  |  |  |  |  | MZR + TAC (1) |
|  |  |  |  | ISA alone | 1 | CyA (1) |
| Relapse 4 | 27 | 17 | (63.0) |  |  |  |
|  |  |  |  | PSL + 1 ISA | 16 | CyA (15), MZR (1) |
|  |  |  |  |  |  |  |
|  |  |  |  | PSL + 2 ISAs | 1 | MZR + TAC (1) |
| Relapse 5 | 19 | 16 | (84.2) |  |  | CyA (11), MZR (2), CYC (1) |
|  |  |  |  | PSL + 1 ISA | 14 |  |
|  |  |  |  |  |  |  |
|  |  |  |  | PSL + 2 ISAs | 2 | CyA + MZR (2) |

Abbreviations: ISA, non-steroidal immunosuppressive agents, CyA; cyclosporine, MZR; mizoribine, TAC; tacrolimus
