# Supplementary material for: Treatment patterns and steroid dose for adult minimal change disease relapses: A retrospective cohort study
Source: PLoS One. 2018 Jun 18;13(6):e0199228. doi: 10.1371/journal.pone.0199228 (PMC6005527; doi:10.1371/journal.pone.0199228)
Supplement: S5 Table — (DOCX) [file pone.0199228.s005.docx]

**S5 Table. Complications and Adverse events in all cases.**

| **All cases (*n*=192)** | | ***n*** | **(%)** |
| --- | --- | --- | --- |
| Complications and  adverse events | Death | 5 | (2.6) |
|  | Infection* | 18 | (9.4) |
|  | Malignancy | 4 | (2.1) |
|  | Thrombosis | 9 | (4.7) |
|  | Cardiovascular disease* | 4 | (2.1) |
|  | Cerebrovascular disease* | 0 | (0.0) |
|  | de novo DM | 25 | (13.0) |
|  | Femoral head osteonecrosis | 7 | (3.6) |
|  | Peptic ulcer disease | 6 | (3.1) |
|  | eGFR 30% decline | 7 | (3.6) |
|  | eGFR 50% decline | 2 | (1.0) |
|  | Maintenance dialysis | 2 | (1.0) |

*required hospitalization

Death: lung cancer (1), lethal arrhythmia (1), bleeding from duodenal ulcer (1),
unknown (1)

Infection: pneumonia (6), cytomegalovirus infection (3), cellulitis (2),
disseminated zoster (2), bacteremia of gram-positive cocci (2), bronchitis (1), nontuberculous mycobacterial infection (1), osteomyelitis (1)

Thrombosis: deep venous thrombosis of lower limb (4), pulmonary embolism (3),
upper limb vein (1), radial artery (1)

Cardiovascular disease: angina pectoris (2), congestive heart failure (1), atrioventricular block (1)

Abbreviations: DM, diabetes mellitus; eGFR, estimated glomerular filtration rate
