# Supplementary material for: Treatment patterns and steroid dose for adult minimal change disease relapses: A retrospective cohort study
Source: PLoS One. 2018 Jun 18;13(6):e0199228. doi: 10.1371/journal.pone.0199228 (PMC6005527; doi:10.1371/journal.pone.0199228)
Supplement: S6 Table — (DOCX) [file pone.0199228.s006.docx]

**S6 Table. PSL dose for the relapse treatment of first relapse as a predictor of second relapse: additional analysis after excluding the patients with very-mild proteinuria.**

| **PSL dose as a categorical variable (High-PSL versus Low-PSL)** | **HR** | **[95% CI]** | **P-value** |
| --- | --- | --- | --- |
|  |  |  |  |
| Univariate | 0.73 | [0.37-1.46] | 0.37 |
| Multivariate ^a)^ | 0.61 | [0.26-1.45] | 0.26 |
| Multivariate with PS ^b)^ after MI ^c)^ | 0.90 | [0.39-2.07] | 0.81 |
| Multivariate with trimmed PS after MI | 0.85 | [0.36-2.35] | 0.73 |
| **PSL dose as a continuous variable (every 10mg/day)** | **HR** | **[95%CI]** | **P-value** |
|  |  |  |  |
| Univariate | 0.78 | [0.56-1.08] | 0.140 |
| Multivariate ^a)^ | 0.80 | [0.51-1.23] | 0.29 |
| Multivariate with PS ^b)^ after MI ^c)^ | 0.77 | [0.54-1.18] | 0.172 |
| Multivariate with trimmed PS after MI | 0.76 | [0.51-1.14] | 0.190 |

Definition: "very-mild proteinuria"; urinary protein level was <1.0 g/gCr at first relapse (n=11; 10 in the Low-PSL group, one in the High-PSL group).

Abbreviations: HR, hazard ratio; CI, confidence interval; PSL, prednisolone; PS, propensity score; MI, multiple imputation

a) adjusted for relapse-free days (every 10 days), serum albumin at first relapse, serum creatinine at first relapse, and urinary protein level at first relapse

b) Propensity score was obtained from the covariates; age (every 10 years), sex (male), relapse-free days (every 10 days), PSL dose before relapse, urinary protein level at first relapse (log transformation) with MI, serum albumin at first relapse with MI, serum creatinine at first relapse with MI, and enhance non-steroid immunosuppressive treatment at the timing of first relapse

c) Multiple imputation was performed for serum albumin, creatinine, and urinary protein (log transformation) using regression model from age (every 10 years), sex (male), relapse-free days (every 10 days), PSL dose before relapse, and non-steroid immunosuppressive agent usage before relapse
