# Supplementary material for: Treatment patterns and steroid dose for adult minimal change disease relapses: A retrospective cohort study
Source: PLoS One. 2018 Jun 18;13(6):e0199228. doi: 10.1371/journal.pone.0199228 (PMC6005527; doi:10.1371/journal.pone.0199228)
Supplement: S7 Table — (DOCX) [file pone.0199228.s007.docx]

**S7 Table. Patient characteristics with or without additional treatment at first relapse.**

|  | **Low-PSL (*n*=36)** | | **High-PSL (*n*=34)** | | **P-value** |
| --- | --- | --- | --- | --- | --- |
| Additional treatment at first relapse | 8 | (22.2) | 7 | (20.5) | 0.87 |
|  | **Additional treatment (+) (n=15)** | | **Additional treatment (-) (n=55)** | | **P-value** |
| Age at first relapse | 60 | [37-67] | 52 | [36-62] | 0.67 |
| Sex, male | 7 | (46.7) | 39 | (70.9) | 0.08 |
| Days to CR at initial treatment | 22 | [12-32] | 17 | [11-27] | 0.35 |
| Relapse free days before first relapse | 396 | [181-627] | 448 | [311-743] | 0.081 |
| Detail of treatment before first relapse |  |  |  |  |  |
| None | 3 | (20.0) | 23 | (41.8) | 0.30 |
| PSL alone | 11 | (73.3) | 29 | (52.7) |  |
| PSL + ISA | 1 | (6.7) | 3 | (5.5) |  |
| Albumin at first relapse, g/dL | 3.8 | [2.9-3.9] | 3.5 | [3.0-3.9] | 0.76 |
| Creatinine at first relapse, mg/dL | 0.81 | [0.63-1.03] | 0.78 | [0.70-0.94] | 0.92 |
| UTP at first relapse, g/gCr | 3.60 | [1.20-10.35] | 2.64 | [1.11-5.20] | 0.59 |
| Days to re-induction of CR | 41 | [21-67] | 14 | [11-27] | <0.001 |

Definition: "additional treatment"; further increasing of PSL dose, added mPSL pulse and/or new non-steroidal immunosuppressants

Abbreviations: CR, complete remission; PSL, prednisolone; ISA, non-steroidal immunosuppressive agents; UTP, urinary protein level
